# Sensing Membrane Stresses by Protein Insertions

**DOI:** 10.1371/journal.pcbi.1003556

**Published:** 2014-04-10

**Authors:** Felix Campelo, Michael M. Kozlov

**Affiliations:** 1 Cell and Developmental Biology Programme, Centre for Genomic Regulation (CRG), Barcelona, Spain; 2 Universitat Pompeu Fabra (UPF), Barcelona, Spain; 3 Department of Physiology and Pharmacology, Sackler Faculty of Medicine, Tel Aviv University, Tel Aviv, Israel; University of Uppsala, Sweden

## Abstract

Protein domains shallowly inserting into the membrane matrix are ubiquitous in peripheral membrane proteins involved in various processes of intracellular membrane shaping and remodeling. It has been suggested that these domains sense membrane curvature through their preferable binding to strongly curved membranes, the binding mechanism being mediated by lipid packing defects. Here we make an alternative statement that shallow protein insertions are universal sensors of the intra-membrane stresses existing in the region of the insertion embedding rather than sensors of the curvature per se. We substantiate this proposal computationally by considering different independent ways of the membrane stress generation among which some include changes of the membrane curvature whereas others do not alter the membrane shape. Our computations show that the membrane-binding coefficient of shallow protein insertions is determined by the resultant stress independently of the way this stress has been produced. By contrast, consideration of the correlation between the insertion binding and the membrane curvature demonstrates that the binding coefficient either increases or decreases with curvature depending on the factors leading to the curvature generation. To validate our computational model, we treat quantitatively the experimental results on membrane binding by ALPS1 and ALPS2 motifs of ArfGAP1.

## Author Summary

Selective targeting of soluble proteins to cellular membranes relies on different mechanisms such as receptor-mediated recruitment or direct binding to specific lipids. A new paradigm has been recently proposed, according to which membrane binding of some proteins is driven by the geometrical and physical properties of the membranes, namely the membrane curvature and lipid packing in the external membrane monolayer. Specifically, several proteins referred to as the membrane curvature sensors have been shown to preferentially bind strongly curved membranes. This mode of protein binding is especially relevant for such fundamental cell processes as endocytosis and carrier generation from ER and Golgi Complex, which involve shaping initially flat membranes into strongly curved ones. A subset of the curvature sensors contains amphipathic or hydrophobic domains that shallowly insert into the membrane. Here we explore computationally the detailed physical mechanism underlying the membrane binding by such proteins and demonstrate that their membrane affinity is not determined by the curvature per se but rather by the membrane stress, independently of the way the stress has been generated. Hence, the significance of our work is in elucidating the relationship between the membrane binding of peripheral proteins carrying shallowly inserting domains and the membrane stresses.

## Introduction

Lipid bilayers serving as matrices of biological membranes bear internal elastic stresses. These stresses can be generated by external forces applied to the membrane surface and driving overall membrane deformations such as generation of membrane curvature and stretching-compression of the membrane area [1], and/or by internal factors such as elastic frustrations, which are intrinsic to the membrane structure [2].

Insertion into the membrane matrix of protein domains spanning completely or partially the lipid bilayer interior must interfere with the intra-membrane stresses. This has to result, on one hand, in the stress-dependence of the energy of the protein insertion into the membrane and, on the other, in alteration of the intra-membrane stresses. The former phenomenon results in the stress sensing by these protein domains, which can be manifested as stress-dependence of the protein partitioning between the membrane and the surrounding aqueous solution [3] and/or as regulation by the stresses of the protein conformational transitions and the related protein activity within the membrane (see e.g. [4]). Alteration of the membrane stress caused by the protein embedding can affect the membrane conformation, e.g. by changing membrane curvature [5], [6].

During the last decade, one of the hot topics discussed in the biophysical literature, and referred to as the curvature sensing by proteins, has been the ability of a number of peripheral membrane proteins to bind preferentially to small liposomes with radii of several tens of nanometers. Commonly for most of these curvature sensing proteins, their binding to membranes has been mediated by shallow insertion into the membrane matrix of an amphipathic or hydrophobic domain [7]. In most cases, such domain is an amphipathic helix [8], but can also be a short hydrophobic loop [9]. The curvature sensing has been demonstrated for numerous proteins involved in intracellular membrane shaping and remodeling such as the N-BAR (Bin-amphiphysin-Rvs) domain-containing protein amphiphysin playing a key role in endocytosis [10]; the GTPase dynamin driving membrane fission [9]; synaptotagmin implicated in membrane fusion [11]; α-synuclein [12]; the lipid droplet enzyme CTP∶phosphocholine cytidylyltransferase (CCT) [13], synapsin I [14], and the autophagosomal protein Barkor/Atg14(L) [15]. A most thorough study of curvature sensing has been performed for a particular kind of amphipathic helices contained in proteins such as Arf1 GTPase-activating protein (ArfGAP1), responsible for the disassembly of the COPI coat [16]–[19]; the golgin GMAP-210; the sterol sensor/transporter Osh4p/Kes1p; and the nucleoporin Nup133 [18]. These helices, which are characterized by bulky amino acids in the non-polar face and small uncharged amino acids in the polar face (mainly serine and threonine), have been demonstrated not only to bind, selectively, to highly curved membranes of small liposomes [16], but also to sense mismatch between the actual membrane curvature and the curvature preferred by the specific lipids composing the outer membrane monolayer [17]. This led to the suggestion that these helices sense membrane curvature by recognizing the curvature dependent defects in lipid packing (see [7], [20], [21] and references therein) and to calling them the amphipathic lipid-packing sensors (ALPS). Since generation and alterations of membrane curvature as well as formation of the lipid packing defects are intimately related to the intra-membrane stresses, it is reasonable to expect that the observed apparent curvature sensing by the insertion-containing proteins is a manifestation of a more general phenomenon of intra-membrane stress sensing.

The goal of the present work is to analyze quantitatively the interplay between the protein insertions imitating amphipathic helices and the membrane stresses produced either by the intra-membrane elastic frustrations or by the external forces leading to different kinds of overall membrane deformations including curvature generation. The major statement of the work is that binding of the insertion-containing proteins to the membrane depends primarily on the local intra-membrane stresses existing within the region of the protein embedding, rather than on the way these stresses have been generated. Particularly, concerning the suggested curvature sensing, we predict that the insertion-containing proteins can exhibit similar binding to membranes of different curvature provided that the membrane stresses in the protein-embedding region are similar. Conversely, these proteins are predicted to bind differently to membranes of similar curvature provided that this curvature is achieved by diverse combinations of the intra- and extra-membrane forces and, hence, corresponds to different intra-membrane stresses. Hence the shallow insertions have to be seen as sensors of the intra-membrane stress rather than the membrane curvature. We substantiate our conclusions by demonstrating that the computational approach we use provides quantitative description of the experimental results on differential binding of ALPS domains to liposomes of various diameters and diverse lipid compositions.

## Model

### Definition of the stress sensing

**Figure 1:**
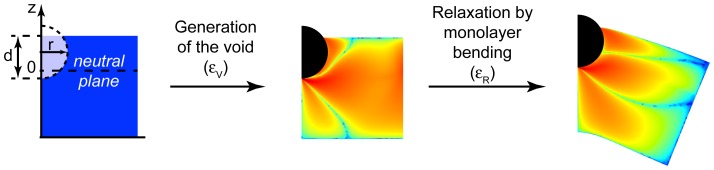
Membrane embedding of a hydrophobic insertion. A cylindrical insertion of a circular cross-section of radius 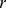 is embedded to a depth 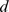 into a lipid monolayer, the neutral plane of the latter lying at two thirds of its thickness (left cartoon). Membrane embedding is presented in two steps. The first step is generation of a void necessary for the insertion embedding without altering the membrane shape. The second step represents the membrane shape relaxation. The color code represents the intra-membrane stresses.

We consider amphipathic helix-like protein domains shallowly embedded into the membrane matrix and refer to these domains as the protein insertions. We model such insertions as cylindrical rods of about one nanometer cross-sectional diameter embedded into the outer membrane monolayer such that the rod axis lies parallel to the membrane plane. The typical embedding depth is about 40% of the monolayer thickness [22] (Fig. 1, left cartoon).

We define as the membrane stress sensing by protein insertions the dependence of the insertion binding to the membrane on the membrane stress. To formalize this definition, we consider a system consisting of *N_p_* protein insertions partitioning between the aqueous solution and the outer monolayers of lipid membranes, which are subject to elastic stresses and can have curved shape. We quantify the insertion binding to the membranes by the binding constant, *K_B_*, defined as a ratio between the number of the insertions remaining in the aqueous solution, 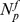, and the number of the membrane bound insertions, 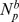,
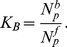
(1)Since the binding constant is measurable experimentally [18], the dependence of *K_B_* on the membrane stress is a convenient quantitative measure of the stress sensing.

The physical reason for the stress sensing is the dependence of the total free energy of the insertion binding, *ε_bind_*, on the membrane stress. The binding energy *ε_bind_*, which is determined as the change of the free energy of the whole system resulting from one insertion binding, has a major contribution, *ε* ^0^, from a number of essential membrane-insertion interactions such as the hydrophobic, hydrogen bonding and electrostatic interactions. On top of that, embedding of the insertion generates intra-membrane strains and the related change of the membrane elastic energy, *ε_el_*, such that,
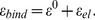
(2)Below, *ε_el_* will be referred to as the elastic binding energy. It follows from thermodynamics of the insertion binding (see Text S1) that for the insertion number, *N_p_*, much smaller than the numbers of water, *N_w_*, and lipid, *N_l_*, molecules, 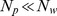, 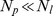, the binding constant can be presented as
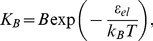
(3)where 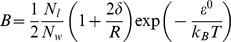, is the stress-independent part of the binding constant, *R* is the radius of the membrane mid-plane and *δ* is the monolayer thickness. The correction 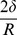 is relevant only if the radius is so small that the difference between the amounts of the lipid molecules in the outer and inner membrane monolayers becomes considerable.

Eq. 3 determines a strong exponential dependence of the binding constant on the elastic binding energy, *ε_el_*, which, in turn, depends on the values of the intra-membrane stresses and their distribution over the membrane thickness.

### Qualitative essence of the elastic binding energy ***ε*** ***_el_***

To reveal the factors that determine the elastic binding energy, *ε_el_*, we dissect the embedding event into two steps. The first step is embedding of the insertion into the membrane while keeping the initial membrane shape unchanged. The variation of the membrane elastic energy at this stage, *ε_V_*, is related to creation of a void in the membrane matrix necessary for the insertion accommodation (Fig. 1). This is accompanied by perturbation of the strains and stresses within the membrane. The second step is a partial relaxation of the stress perturbation due to the change of the membrane shape, which is accompanied by another change of the elastic energy, *ε_R_* (Fig. 1).

The energy of the first step, *ε_V_*, can be seen as thermodynamic work performed against the membrane stresses in the course of the void generation, which can be presented as a sum of two contributions,
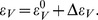
(4)In essence, 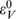 is the work of the void formation performed against the initial stresses existing in the membrane before insertion, while 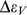 accounts for the energy of the stress perturbation.

Summarizing, the elastic binding energy can be presented as consisting of three contributions
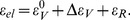
(5)


As shown below, in all relevant cases 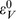 represents the major part of the energy of void formation. In addition, 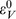 turns out to be the most convenient value accounting for the distribution of the initial unperturbed stresses in the context of the void formation. Therefore, we will refer to 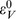 as the void energy and use it as a variable characterizing the stressed state of the membrane before insertion.

Since the protein insertions we are considering do not span the whole membrane but rather get shallowly embedded into the membrane matrix, the total energy of the void formation, *ε_V_*, and hence the elastic binding energy *ε_el_*, depend on the character of the stress distribution through the membrane thickness. This distribution can be described by the trans-membrane stress profile *σ*(*z*) [2] (Fig. S1). In Text S1 we discuss the model assumptions concerning the properties of the trans-membrane stress profile and the relationships between *σ*(*z*) and the overall force factors determining the membrane stressed state, namely, the lateral tension *γ* and the bending moment *τ*. These determine the ways of the stress profile generation by application to the membrane of external forces or by changing the monolayer spontaneous curvatures through variations of their lipid compositions (see for review [23], [24] and references therein). Specifically, the void energy representing, as mentioned above, the thermodynamic work against the initial stresses needed for the void formation, can be related to the initial stress profile 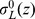 existing within the outer membrane monolayer before the insertion embedding,
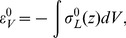
(6)where the integration is performed over the volume of the void.

Whereas, according to Eq. 6, the void energy will be calculated by a direct integration of the initial stress profile, the additions to the energy related to the emerging strains, 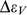, and the energy of relaxation *ε_R_*, require a more involved numerical computation including determination of the relaxed membrane shape and will be performed based on the relationships Eqs. S10–S13 using Comsol Multiphysics [5].

### The ways of stress generation

We address the sensing by the protein insertions of the intra-membrane stress generated by several specific ways that are experimentally feasible and biologically relevant.

**Figure 2:**
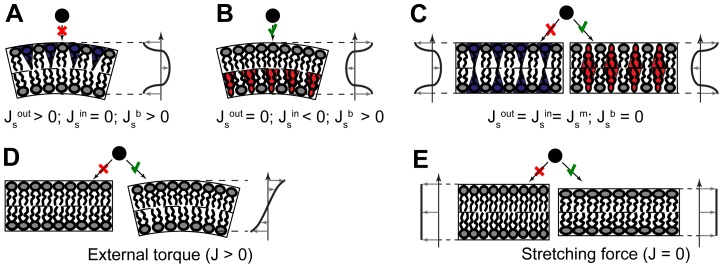
Five ways of generating membrane stress preceding the insertion embedding. A qualitative trans-monolayer stress profile, incorporating the main characteristics, is shown for illustrative purposes. (A) Addition of lipids with inverse conical molecular shape (lysolipids, depicted in blue) to the outer monolayer. The induced positive monolayer spontaneous curvature of the outer monolayer, 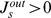, results in a positive bilayer curvature 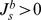 and the corresponding trans-membrane stress profile. (B) Insertion of molecules with conical molecular shape (e.g. DAG, depicted in red) to the inner monolayer. The corresponding negative monolayer spontaneous curvature 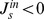 induces a positive bilayer curvature 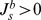 and the corresponding trans-membrane stress profile. (C) Symmetric enrichment of the two membrane monolayers in inverted conical (left cartoon) or conical (right cartoon) lipids. The bilayer remains flat, 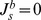, but the trans-membrane stress profile develops. (D) Membrane bending by the action of an externally applied torque that induces a positive bilayer curvature *J*>0 and the corresponding trans-membrane stress profile. (E) Membrane stretching (right cartoon) or compression (left cartoon) by external force and the corresponding trans-membrane stress profiles. In all panels, red crosses and green ticks illustrate, respectively, low and high binding affinities of protein insertions for differently stressed membranes.

First, we consider the stress resulting from the spontaneous curvature *J_s_*, which is produced in the membrane monolayers by changing the monolayer lipid composition (see Text S1) and consider three different situations:

- A spontaneous curvature, 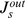, is generated in the outer membrane monolayer exposed to embedding of the insertion, whereas the properties of the inner monolayer do not change (Fig. 2A). As a result, the outer monolayer tends to bend, while the inner monolayer resists this bending. Assuming that the two monolayers have equal bending rigidity *κ*, and that the monolayer spontaneous curvature *J_s_* is much smaller than the inverse monolayer thickness, the whole membrane adopts a bent shape with curvature 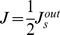. As a result, the outer and the inner monolayers develop a bending moment
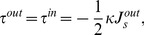
(7)with the corresponding trans-monolayer stress profile (Fig. 2A) (Text S1).
- A spontaneous curvature, 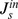, is generated in the inner monolayer, while the elastic properties of the outer monolayer remain unchanged (Fig. 2B). In this case the whole membrane bends assuming a shape with curvature 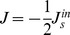 and trans-monolayer stress profiles generated in the two monolayers correspond to the bending moments (Fig. 2B)
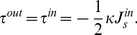
(8)
- A spontaneous curvature, 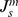, is induced in both the outer and inner monolayers (Fig. 2C). In this case, the bilayer remains symmetric and does not bend, while each monolayer develops a stress-profile with a bending moment (Fig. 2C),
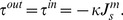
(9)

Second, we consider application of an external torque to an initially flat bilayer, which results in generation of a bilayer curvature *J* (see Text S1). This corresponds to the experimental procedures of generation of small liposomes *in vitro* by means of sonication or extrusion. The external torque produces in the outer monolayer a trans-monolayer stress profile with a bending moment
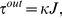
(10)while the stress-profile in the inner monolayer corresponds to a bending moment (Fig. 2D).
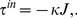
(11)


Finally, we analyze the case where the membrane stress is produced by applying a stretching force to the flat monolayer, which generates an overall lateral tension, *γ*, related to the trans-membrane stress profile (see Text S1) (Fig. 2E). Such a force can be produced as a result of, e.g., osmotic stretching of the liposomal membrane.

### Statement of the problem

We model the membrane as consisting of two monolayers each characterized by a bending modulus of 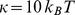 [25]. The monolayers can be laterally uncoupled, meaning that there is a reservoir of material for each monolayer with which the lipid molecules can be exchanged. This is the case in most of the biologically relevant situations where the insertions are restricted to a small membrane patch for which the surrounding membrane plays a role of a lipid reservoir. Alternatively, the monolayers can be laterally coupled if, e.g., some rigid barrier restricts the lipid exchange between the membrane patch accommodating the insertions and the surrounding membrane, or if the proteins are recruited to the entire area of a closed membrane, so that there are no lipid reservoirs to exchange with, as it occurs in common *in vitro* assays.

An insertion is modeled as a rigid cylindrical rod with a radius of 0.5 nm that partially embeds into the outer membrane to a depth of 0.8 nm, which imitates the typical size and insertion depth of amphipathic helices [22]. In general, we consider the length of the insertion along the membrane plane to be 2 nm, characteristic for some amphipathic helices [22], [26].

Our goal is to describe quantitatively the membrane stress sensing by insertions in all five above-mentioned cases of membrane stress generation. We will compute the dependence of the insertion binding constant, *K_B_*, Eq. 3, on the void energy, 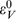, Eq. 6. The absolute value of the binding constant depends, according to Eq. 3, on the stress-independent factor *B*, whose value is unknown since it accounts for a combination of the electrostatic, hydrophobic and hydrogen-bonding interactions between the membrane and the insertion, 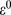 , (Eq. 2). We will therefore compute the relative binding constant, 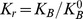 where 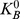 characterizes the insertion embedding into the initial unstressed flat membrane. The relation between 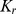 and the elastic binding energy is
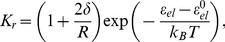
(12)where 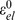 is the elastic binding energy prior to the stress generation and *R* is the curvature radius of the membrane in the stressed state under assumption that the membrane shape is spherical.

For the cases where generation of the membrane stress is accompanied by membrane curvature variations, we will illustrate the relationship between the stress sensing and the earlier suggested curvature sensing by presenting the binding constant, 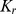, as a function of the curvature.

### Computational method

The equilibrium distributions of the membrane stresses and strains before and after insertion embedding have been found by solving the set of partial differential equations for the displacement field, 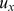 and 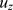, as explained in [5]. Briefly, for the case of two-dimensional deformations where the membrane adopts a tubular shape with the y-axis laying along the tube, the equations to be solved are
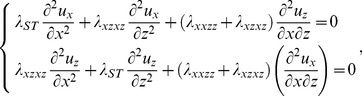
(13)where 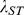, 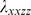, and 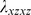 are the trans-monolayer profiles of the monolayer elastic moduli [5], [27]. This approach accounts in a continuous manner for variations of the local pressures and elasticities at distances of sub-nanometer scale. This is equivalent to usage of intra-membrane force field for modeling membrane processes by molecular dynamic simulations, which proved to provide a quantitative description of the membrane behavior. The implemented trans-membrane pressure and elasticity profiles represent a simplified version of those computed recently by the state-of-the-art molecular dynamic simulation using Martini force field [27]. Therefore, our predictions are expected to be of at least semi-quantitative accuracy. For a further discussion about the advantages and disadvantages of both continuum and simulation approaches see Ref. [28].

The equations (Eq.13) were solved for a membrane element of length *L* and thickness 2*h*, where *h* is the monolayer thickness, with the following boundary conditions. First, the insertion is assumed to be much more rigid than the lipid bilayer and hence imposes a horizontal displacement that corresponds to the insertion shape. Second, the top and bottom surfaces of the bilayer are set free, implying that the stresses 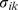 vanish there. Finally, the right boundary for each monolayer is a symmetry plane, which remains straight but can rotate with respect to the left boundary and can also have a certain constant displacement in both the horizontal and vertical directions. The rotation angle and the displacements are found from minimization of the elastic free energy change upon insertion.

The set of equations (Eq.13) was solved by a finite element method scheme using the commercial software Comsol Multiphysics, allowing one to represent the membrane deformation, calculate the elastic free energy change upon insertion, as well as the void energy. The membrane shape was discretized for the finite element method using a triangular mesh starting with at least 1908 elements, and refined using and adaptive mesh refinement to at least 5514 elements.

For simulation of an initial membrane stress created by a combination of different monolayer spontaneous curvatures, the lateral stress profile has been taken according to Eq. S17. For simulation of the application of an external torque, a constant torque has been applied to the right boundary of the bilayer for both laterally coupled and uncoupled monolayers. Finally, a constant force perpendicular to the right boundary has been applied to simulate the case of a stretched or compressed membrane. In all these cases, the free energy minimization has been acquired by taking into account the work of deformation produced by the externally applied forces.

## Results

### Stress sensing in case of laterally uncoupled monolayers

**Figure 3:**
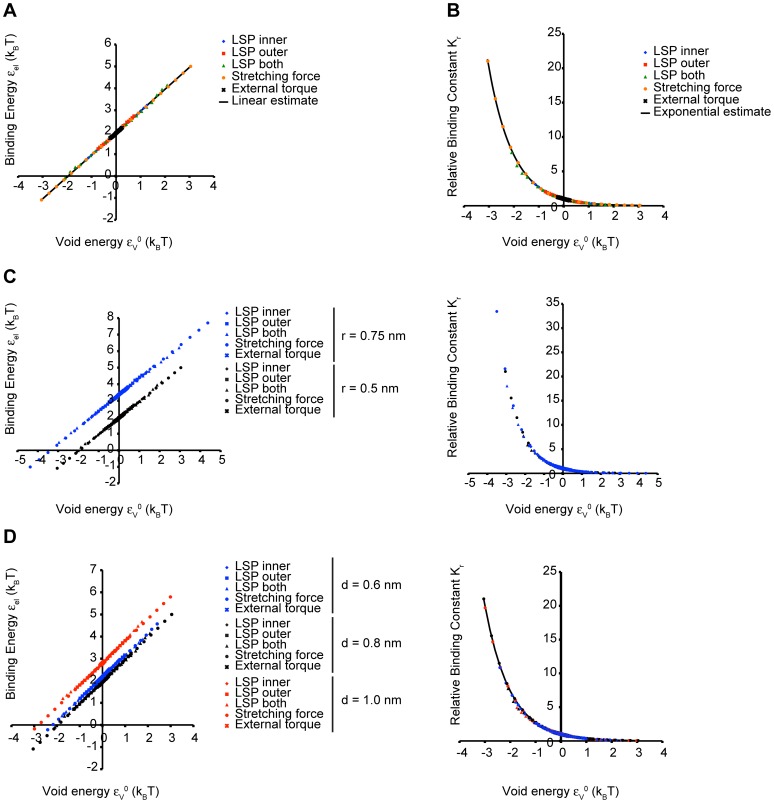
Stress sensing by hydrophobic insertions for laterally uncoupled monolayers. The elastic binding energy 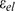 of insertion was computed using the elastic model of a lipid bilayer for the insertion length of 2 nm, the insertion cross-sectional radius of 0.5 nm and the insertion embedding depth of 0.8 nm, as it has been estimated based on structural data for typical amphipathic helices. The monolayer thickness is taken to be 2 nm. The membrane stress was generated in five ways, as presented in Fig. 4 below. (A) The elastic binding energy 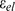 as a function of the void energy 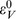, all points laying approximately on a straight line of slope one (black line). (B) The relative binding constant 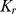 as a function of the void energy 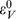. The black line shows the expected exponential profile. (C) The elastic binding energy 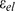 (left) and the relative binding constant 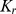 (right) as functions of the void energy 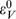 for two different physiologically relevant cross-sectional radii (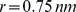 in blue, and 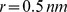 in black) and the embedding depth 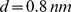. (D) The elastic binding energy 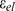 (left) and the relative binding constant 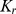 (right) as functions of the void energy 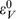 for the insertion cross-sectional radius 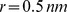 and different biologically feasible values of the embedding depth (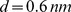 in blue, 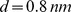 in black, and 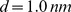 in red).

The computed dependences of the elastic binding energy, 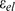, and the relative binding constant, 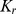, on the void energy, 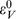, for all five aforementioned scenarios of the stress generation are presented in Fig. 3.

Remarkably, the results for 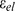 obtained for different ways of the stress generation collapse to a single straight line with a slope equal to one (Fig. 3A). This infers, based on Eq. 5, that the contributions from the stresses emerging in the course of insertion, 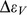, and the shape relaxation, 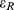, have a negligibly small dependence on 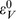. While the void energy 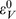 is determined solely by the intra-membrane stresses preceding the insertion, 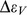 and 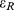 are expected to depend on the scenario of the stress generation. Although 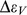 is expected to be a small correction to the binding energy, the shape relaxation part of the binding energy, 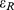, is of the same order of magnitude as the void energy 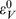. Our results show that the shape relaxation is independent of the stress distribution along the membrane in the initial state. As a result, the elastic binding energy 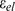 is practically independent of the way the stress is produced.

Also the dependences of the relative binding constant, 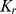, on the void energy, 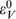, computed for the five scenarios of the stress generation collapse to a unique curve described by the exponential function 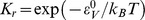 (Fig. 3B). According to Eqs. 12 and 5, this is the result of the above-obtained negligibility of the dependences of the energies 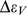 and 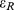 on the void energy, 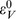, and of the smallness in most cases of the ratio between the membrane thickness and the curvature radius, 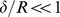. Hence, also the relative binding constant, 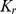, quantifying the stress sensing does not depend on the scenario of stress generation.

Amphipathic helices of different proteins have various dimensions and could, potentially, get embedded to different depths into the lipid monolayer matrix. Insertion induced curvature depends substantially on the insertion size and the embedding depth [5], which indicates that also the elastic binding energy, 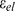, and, hence, the relative binding constant, 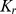, may depend on these parameters.

Fig. 3C presents a comparison of the computed dependences of 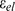 and 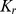 on the void energy, 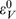, for insertions with cross-sectional radii of 0.75 nm and 0.5 nm for the five ways of stress generation. Both types of insertions are assumed to be embedded to the same depth of 0.8 nm, and have the same length of 2 nm. Fig. 3D shows the results obtained for different embedding depths. In both cases, whereas the values of the elastic binding energy do depend on the insertion radius (Fig. 3C, left panel) and embedding depth (Fig. 3D, left panel), the variation of 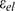 as a function of 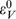 is always represented by a straight line with slope equal one. This means that although the stress-independent part of the elastic binding energy varies with the insertion size and the embedding depth, the stress-dependent part does not and is, practically, equal to 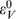. As a result, the dependence of the relative binding constant, 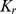, on the void energy, 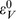, and, hence, the stress sensitivity are independent of the cross-sectional radius of the insertion (Fig. 3C, right panel) and of the embedding depth (Fig. 3D, right panel).

Summarizing, the protein insertions are predicted to be universal sensors of membrane stresses existing in the region of the insertion embedding.

### Relationship between insertion binding and membrane curvature

**Figure 4:**
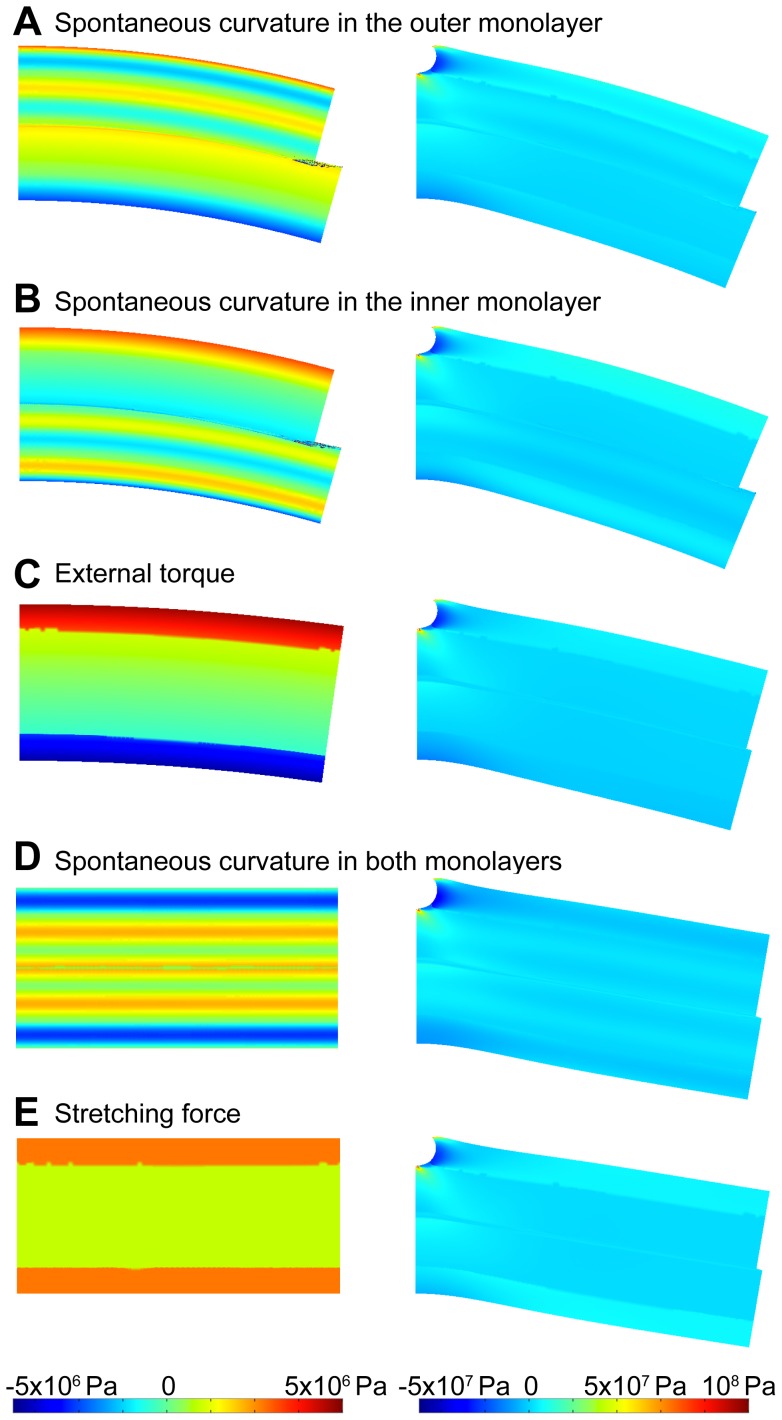
Computed membrane shapes for different ways of stress generation before (left column) and after (right column) the insertion embedding. (A) Changing the spontaneous curvature of the outer monolayer; (B) Changing the spontaneous curvature of the inner monolayer; (C) Application of an external torque; (D) Symmetric generation of the spontaneous curvature of both monolayers; (E) Action of an externally applied stretching force. The color code represents the value of the lateral stress profile 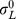 at each point of the membrane in all panels (different scales on left and right panels).

In three out of five considered scenarios of the stress generation, building up of the stress is accompanied by emergence of membrane curvature. Fig. 4 presents examples of the computed shapes, which are adopted by an initially flat bilayer as a result of the stress generation (left panels) followed by the insertion embedding (right panels). If the stress is produced as a consequence of inducing the spontaneous curvature in the outer (Fig. 4A) or inner (Fig. 4B) monolayer, or by application of an external torque (Fig. 4C), the membrane acquires curvature prior to the insertion embedding such that the insertion interacts with a bent membrane. In case the stresses result from the spontaneous curvature induced symmetrically in both monolayers (Fig. 4D), or from an overall membrane stretching (Fig. 4E), the insertions get embedded into a flat membrane, whereas the curvature builds up only at the latest stage as a result of the shape relaxation.

Since, as emphasized in the Introduction, an extended literature has been devoted to the curvature sensing by proteins, we show here the correlation between the strength of the insertion binding and the membrane curvature existing prior to the insertion embedding for the three relevant scenarios of the stress generation (Fig. 4A–C).

**Figure 5:**
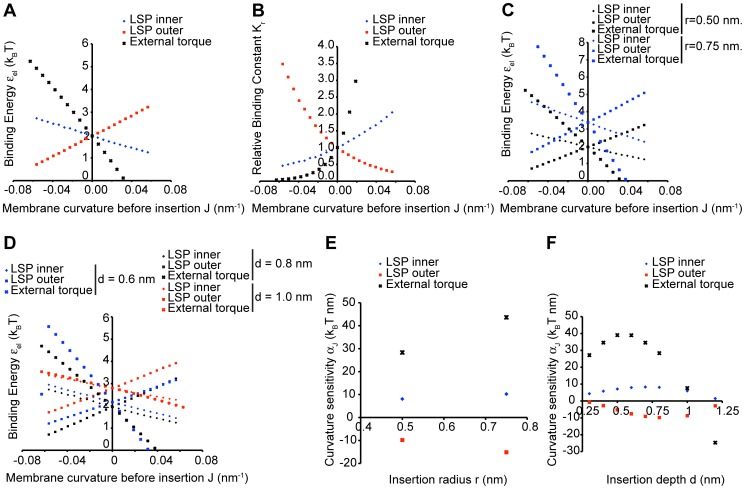
Insertion binding as a function of the initial membrane curvature for the three scenarios of stress generation accompanied by creation of membrane curvature. The monolayers are laterally uncoupled. The elastic binding energy 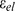 (A) and the relative binding constant 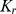 (B) are presented as functions of the membrane curvature before insertion *J*. (C) The elastic binding energy 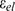 as a function of the membrane curvature *J* for insertions of different cross-sectional radii (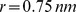 in blue, and 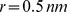 in black) embedded to the depth 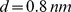. (D) The elastic binding energy 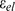 as a function of the membrane curvature *J* for insertions of the cross-sectional radius 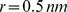 embedded to three different depths (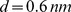 in blue, 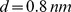 in black, and 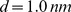 in red). (E–F) The curvature sensitivity 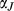 as a function of the insertion radius *r* (E) and the insertion embedding depth *d* (F).

Fig. 5A,B presents the dependence of the elastic binding energy, 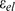, and the relative binding constant, 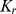, on the pre-insertion membrane curvature, *J*. We performed our analysis for a large range of membrane curvatures in order to take into account the highly curved membranes generated, e.g., during transport carrier formation and endocytosis. If the stress is generated by inducing the spontaneous curvature of the inner monolayer or by application of an external torque, the elastic binding energy, 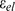, decreases and the binding constant, 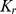, increases with the curvature. Thus, the insertion binding is predicted to be stronger for small rather than large liposomes, in agreement with the experimental results of ALPS binding [17]. Opposite prediction corresponds to the case where the stresses are produced by the spontaneous curvature generation in the outer monolayer. In this situation, 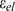, increases and the binding constant, 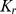, decreases with growing curvature meaning that the insertions are expected to bind stronger to large rather than to small liposomes. These results can be qualitatively understood by considering the stresses in the external part of the outer monolayer for the different scenarios of curvature generation and their influence on the elastic binding energy 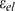.

Hence, the dependence of the insertion binding on the membrane curvature and, therefore, the apparent curvature sensing, depend on the way the curvature is produced and can be opposite for different scenarios of curvature generation.

It is convenient to quantify the apparent curvature sensitivity by the slope, 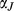, of the line representing, approximately, the elastic binding energy 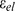 as a function of the membrane curvature *J*, so that 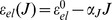. Following Eq. 12, and under the assumption of smallness of the membrane curvature with respect to the inverse monolayer thickness, 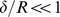, the relative binding constant can be then expressed as 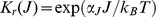. Positive values of the curvature sensitivity, 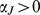, correspond to preferable insertion binding to membranes with larger curvature (small liposomes), while negative curvature sensitivity, 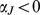, means preferable binding to membranes with smaller curvature (large liposomes).

Based on the results above, the sign of the curvature sensitivity 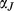 is determined by the way the membrane curvature is produced. The absolute value of the curvature sensitivity 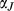 depends on the insertion cross-sectional radius and the embedding depths. These dependences are presented in Fig. 5E,F for the three ways of stress generation leading to membrane curvature. The model predicts that the absolute value of the curvature sensitivity 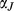 increases with the insertion cross-sectional radius (Fig. 5E). Interestingly, 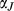 is predicted to change non-monotonously as a function the embedding depth (Fig. 5F). It reaches a maximum for the positive, 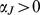, and minimum for the negative, 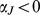, values of the curvature sensitivity at some intermediate embedding depths, the latter varying between the different ways of curvature generation (see Fig. 5F).

Summarizing, the protein insertions cannot be considered as universal curvature sensors since the character of the curvature sensing depends on the specific curvature generating factors.

### Analysis of the experimental results on membrane binding by ALPS motifs

To validate our proposal of the intra-membrane stress sensing by protein insertions, we used the suggested computational model to treat the quantitative experimental results on membrane binding by the two ALPS motifs of ArfGAP1, ALPS1 and ALPS2, which fold within membranes into amphipathic helices. These studies address the dependence of the ALPS binding on the liposome radius [19] and lipid composition [29].

The quantity measured in [19] was the percentage of ALPS1 and ALPS2 amphipathic helices bound to liposomes of 34 nm, 42 nm, and 90 nm radii. Based on these data, we first found the values that can be obtained from the experimental data and accessible to determination by our model. The absolute values of the binding constant, 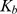, are unaccessible, since they depend on the part of the binding energy, 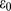 , accounted by the parameter *B* (see Eq. 3) that is not stress-dependent but rather determined by a combination of strong interactions such and hydrophobic, electrostatic and hydrogen-bonding interactions. However, we can eliminate the unknown stress-independent parameter *B* by using the relative binding constants 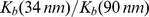 and 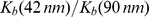, presented in Table 1, and compare them with the experimental results.

**Table 1:** Relative binding constants of ALPS1 and ALPS2 to liposomes of different sizes.

Amphipathic helix	K_b_(34 nm)/K_b_(90 nm)	K_b_(42 nm)/K_b_(90 nm)
ALPS1	46.75	11.51
ALPS2	5.33	2.25

To obtain the corresponding values computationally, we took into account several aspects of the experimental system [19]. First, the liposomes were formed by extrusion or sonication meaning that the bending moment and the corresponding curvature, *J*, were generated by an external torque applied to the membranes. Second, the protein motifs were inserted along the whole membrane area rather than locally [19]. Therefore, the liposome monolayers must be seen as laterally coupled since they could not exchange lipid molecules with any lipid reservoir. The modifications of the computational method needed to account for the monolayer coupling were introduced in [5]. Finally, the length and, especially, the embedding depth of ALPS1 and ALPS2 amphipathic helices could be estimated based on structural data but have not been precisely determined and, therefore, had to be considered as fitting parameters.

**Figure 6:**
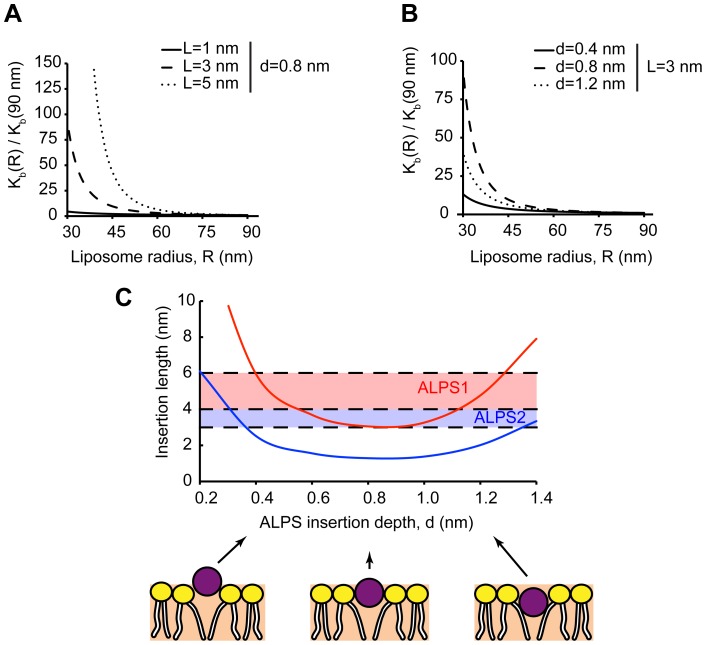
Treatment of experimental data on binding of ArfGAP1 ALPS motifs to liposomes of different sizes. (A) The relative binding constant 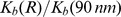 was numerically computed as a function of the liposome radius for laterally coupled monolayers bent by the action of an externally applied torque, and plotted as a function of the liposome radius *R* for the insertion cross-sectional radius 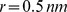, the insertion depth 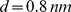, and three different insertion lengths (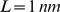, solid line; 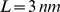, dashed line; and 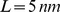, dotted line). (B) The same quantities as in (A) for a 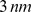 long insertion embedded to three different depths (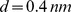, solid line; 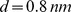, dashed line; and 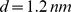, dotted line). (C) The optimal insertion length, *L*, as a function of the insertion embedding depth, *d*, that best fits the experimental results presented in Table 1 for both ArfGAP1 ALPS1 (red line) and ALPS2 (blue line). The shaded regions represent the range of ALPS lengths estimated for each motif based on structural data.

Fig. 6 presents the dependences of the computed binding constant ratio 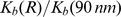 on the liposome radius *R* for different insertion lengths (Fig. 6A) and embedding depths (Fig. 6B). The cross-sectional radii of the amphipathic helices were taken to be 0.5 nm for all computations. Fitting the computed values (Fig. 6A,B) to those derived from the experiments (Table 1), we find for each ALPS motif the relationship between the length and embedding depth guaranteeing a quantitative agreement between the experimental and theoretical results (Fig. 6C). The expected lengths of the amphipathic helices, estimated based on the structural data, vary between 4–6 nm for ALPS1 and 3–4 nm for ALPS2 [19] as presented by the shaded region in Figure 6C. Comparison of the computed and the expected values (Fig. 6C) predicts that ALPS1 and ALPS2 amphipathic helices embed to a depth close to 0.4 nm with a tendency of ALPS1 to penetrate the membrane a little deeper than ALPS2.

In [29] the ArfGAP1 ALPS binding was studied in dependence on the membrane lipid composition, which was modified by symmetric addition to the two membrane monolayers of diacylglycerol (DAG) and phosphatidylethanolamine (PE), the lipids generating a negative monolayer spontaneous curvature [23]. The percentage of the membrane bound ArfGAP1 was measured as a function of the mole fraction of these lipids within the membrane. As explained above (see also Text S1) and according to Eq. 9, symmetric generation of spontaneous curvature of the membrane monolayers leaves the membrane flat but produces stresses in each monolayer. These stresses are expected to modulate the amphipathic helix binding.

**Figure 7:**
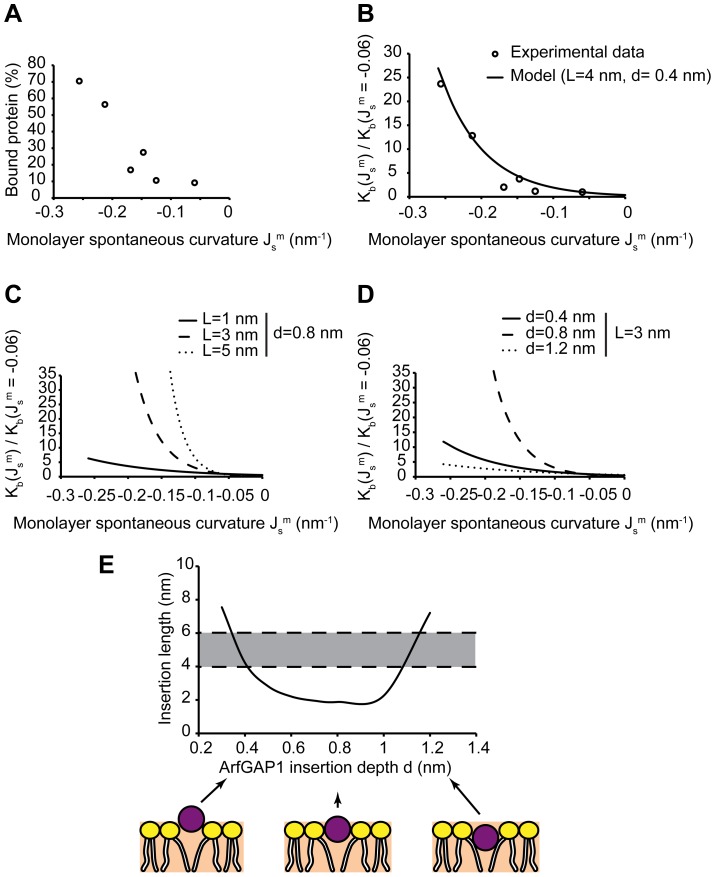
Treatment of experimental data on the binding of ArfGAP1 to liposomes of different lipid compositions. (A) The fraction of bound ArfGAP1 to liposomes of different lipid composition as a function of the estimated monolayer spontaneous curvature 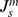, as taken from the experimental study [29] (see Table 2). (B) The relative binding constant as a function of the monolayer spontaneous curvature 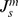, as taken from the experimental study [29] (circles), and a comparison with a fit using our model, for an insertion length of 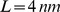 and a depth of insertion of 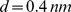
(solid line). (C) The relative binding constant numerically computed for laterally coupled symmetric monolayers as a function of the monolayer spontaneous curvature, 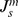, for the insertion cross-sectional radius 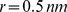, the insertion depth 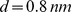, and different insertion lengths (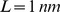, solid line; 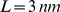, dashed line; and 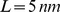, dotted line). (D) The same quantities as in (C) plotted for a 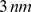 long insertion embedded to three different depths (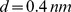, solid line; 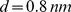, dashed line; and 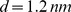, dotted line). (E) The optimal insertion length, *L*, as a function of the insertion embedding, *d*, that best fits the experimental results presented in Table 2 for ArfGAP1 (solid line). The shaded region represents the estimated range of ArfGAP1 insertion lengths.

To enable the comparison of the experimental results [29] with the model predictions, we first plot the measured fraction of bound protein as a function of the monolayer spontaneous curvature, 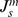, (Fig. 7A). The latter is assumed to be related to the monolayer lipid composition by the relationship, 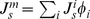, where 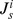 and 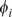 are, respectively, the spontaneous curvature and the intra-monolayer area fraction of the individual lipid components and the summation is performed over all lipid components [30]. The area fractions of the constituent lipids are taken from [29], while the lipid spontaneous curvatures are taken to be 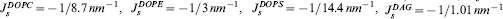 (see [23] and references therein).

A convenient quantity to be derived from the experimental data is the ratio between the protein binding constants for certain monolayer spontaneous curvatures 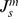, and for the background spontaneous curvature of 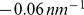 corresponding to 70% PC and 30% PS. The values of this ratio in dependence on 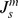 are presented in Table 2 and Fig. 7B. The same ratio of the binding constants was computed based on our model using the insertion length and the embedding depth as fitting parameters and assuming, as mentioned above, that the monolayers are laterally coupled. The relationship between the ArfGAP1 insertion length and its embedding depth that best fits the experimental data in Fig. 7B is presented in Fig. 7E where the shaded region corresponds to the feasible values of these parameters. According to these results, for a realistic total insertion length of 4 nm, the required embedding depth is about 0.4 nm, which is consistent with the above estimations (Fig. 6).

**Table 2:** Liposome monolayer spontaneous curvatures and relative binding constants of ArfGAP1 to liposomes of different lipid compositions.

DOPC∶DOPE∶DOPS∶DAG	J_s_^m^ (nm^−1^)	K_b_(J_s_^m^)/K_b_^0^
70∶0∶30∶0	−0.060	1
60∶0∶30∶10	−0.147	3.76
40∶30∶30∶0	−0.125	1.17
30∶30∶30∶10	−0.213	12.82
20∶50∶30∶0	−0.169	2.02
10∶50∶30∶10	−0.256	23.67

## Discussion

It has been proposed and extensively discussed in the literature that some peripheral membrane proteins are able to sense large membrane curvatures [7]. In the experimental studies devoted to verification of this idea, the curvature sensing was manifested by a preferential binding of such proteins to small liposomes of few tens of nanometer radii [17], [31]. The reason for the attractiveness of the concept of curvature sensing by proteins is a straightforward and, therefore, feasible mechanism it suggests for interplay between the geometry and protein composition of cell membrane patches. Such interplay including a positive feedback between the membrane bending and the local protein concentration may have far reaching consequences for the mechanisms of such intra-cellular processes as endocytosis [32] and generation of intra-cellular membrane carriers from the endoplasmic reticulum and the Golgi complex [33], which involve membrane shaping and remodeling by proteins [34].

Two classes of protein domains have been proposed to sense membrane curvature: hydrophilic intrinsically curved domains, such as BAR domains, able to bind the membrane surface and referred to as the membrane scaffolds [10]; and small amphipathic or hydrophobic domains, such as amphipathic *α*-helices, which get shallowly embedded into the lipid monolayer matrix and are referred to as the hydrophobic insertions [16].

The potential importance of the curvature sensing by proteins raises a question about the mechanism of this phenomenon. The mechanism by which the protein scaffolds sense membrane curvature is straightforward and related merely to the membrane bending energy. The closer the membrane curvature is to that of the scaffolding protein domain, the less membrane bending deformation is required for the protein attachment to the scaffold and, hence, the less bending energy is consumed for the attachment event making it more energetically favorable. Hence, the scaffolding protein domains must sense the membrane curvature per se.

The situation with the hydrophobic insertions, which are not characterized by a curved shape and penetrate the membrane interior rather than stick to the membrane surface, appears to be more complicated. The mechanism of curvature sensing by the insertions has to be related to the internal membrane stresses, which can arise from various membrane deformations rather than, solely, from the overall membrane bending. Here we analyzed numerically the changes of the membrane elastic energy related to the insertion embedding with a goal to understand whether the insertions sense, indeed, the membrane curvature per se, or, alternatively, they sense the intra-membrane stresses independently of the way the stresses are generated.

A protein domain can be considered as a curvature sensor per se if its binding to the membrane is influenced by the membrane curvature, and the curvature-dependence of the binding coefficient is the same for different ways of the membrane curvature generation. Our calculations showed that this is not the case for the protein domains, such as amphipathic *α*-helices, which get shallowly inserted into the membrane matrix. As illustrated in Fig. 5A the binding constant of such domains increases with increasing curvature for the cases where the curvature is produced by an externally applied torque (black asterisks), or by addition of lipids with negative spontaneous curvature to the inner monolayer (blue tilted squares), but decreases if the curvature is produced by addition of lipids with a positive spontaneous curvature to the outer monolayer (red squares). Hence the curvature sensing is not a universal property of the protein insertions.

At the same time, according to our model, the protein insertions are universal sensors of the intra-membrane stresses within the region of the insertion embedding. The dependence of the insertion binding coefficient on these stresses does not depend on the way the stresses are generated (Fig. 3A). The mechanism of this stress sensing is based on the elastic energy coming from formation of a void in the membrane matrix necessary to accommodate the insertion (Fig. 1). The thermodynamic work of the void formation is performed against the internal stress existing within the membrane matrix, which is equivalent to an intra-membrane pressure (taken with opposite sign). As a result the mechanism of the stress sensing by hydrophobic insertions can be seen as a “pushing the walls” mechanism.

It has to be noted that the stress-sensing mechanism must underlie also the curvature sensing by transmembrane proteins spanning the whole membrane thickness [35]. Distribution of transmembrane proteins between different regions of the same membrane must be determined by the thermodynamic work, which has to be performed against the intra-membrane stresses in order to create a void accommodating the protein. In case this work varies along the membrane, the transmembrane proteins must partition accordingly. A specific example of such situation is lateral partitioning of trans-membrane proteins characterized by asymmetric cone-like effective shapes along membranes with varying curvature [35] or surface concentration of non-bilayer lipids. The difference between trans-membrane proteins and shallow insertions is that the curvature sensitivity by the former cannot be related to the protein binding coefficient since such proteins are very hydrophobic and, therefore, insoluble in aqueous solutions.

The suggested mechanism changes considerably the view on the potential role of proteins domains serving as hydrophobic insertions in the protein targeting and the mode of their action in membrane shaping processes. Further in vitro experimentation aimed at quantitative characterization by biochemical methods of binding of different proteins containing hydrophobic insertions to liposomes of different lipid composition, curvature, or membrane tension, would provide stronger evidence of the stress-sensing mechanism proposed here.

## Supporting Information

Figure S1
**Qualitative essence of the trans-monolayer lateral stress profile.** Cartoon of a lipid monolayer showing the lateral stress occurring at the level of the polar headgroups, 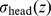, and of the acyl chains, 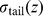, for a flat monolayer (A) or a positively bent 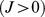 monolayer (B).(TIF)

Figure S2
**Lateral stress profile used in the computations.** The depth modulation of the monolayer lateral stress profile 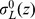 used in the numerical computations as a result of varying the lipid composition is presented for a negatively curved monolayer with a spontaneous curvature of 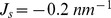.(TIF)

Text S1 **This file contains detailed description of the model.** Thermodynamic model of protein binding to membranes. Properties and the ways of generation of trans-membrane stress profile.
(PDF)
