# Supplementary figures and images for: Sensing Membrane Stresses by Protein Insertions
Source: PLoS Comput Biol. 2014 Apr 10;10(4):e1003556. doi: 10.1371/journal.pcbi.1003556 (PMC3983069; doi:10.1371/journal.pcbi.1003556)

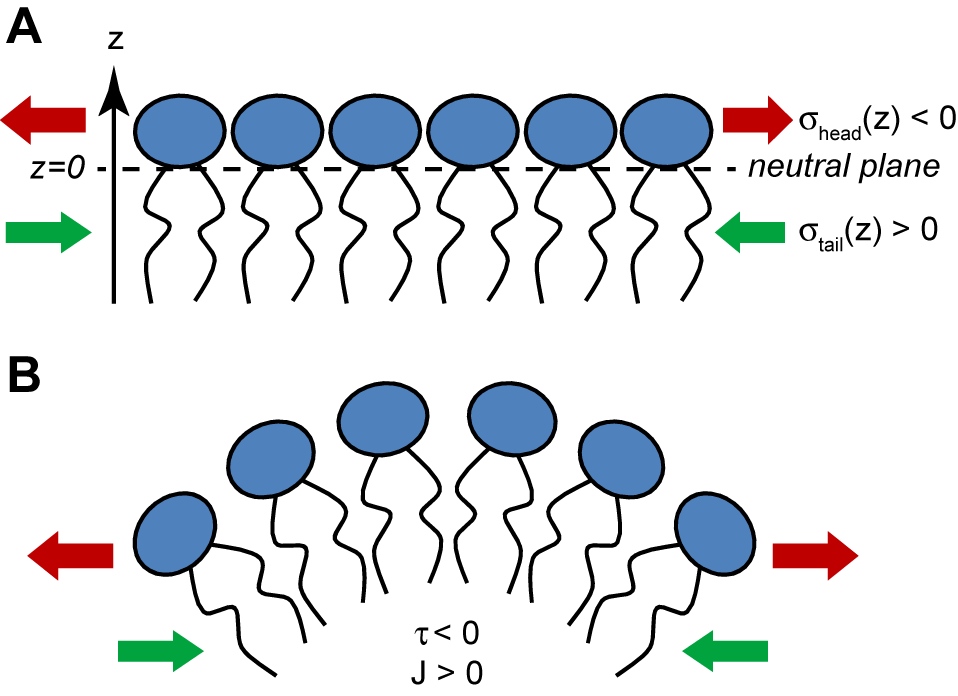

Supplement: Figure S1 — Qualitative essence of the trans-monolayer lateral stress profile. Cartoon of a lipid monolayer showing the lateral stress occurring at the level of the polar headgroups, , and of the acyl chains, , for a flat monolayer (A) or a positively bent monolayer (B). (TIF) [file pcbi.1003556.s001.tif]

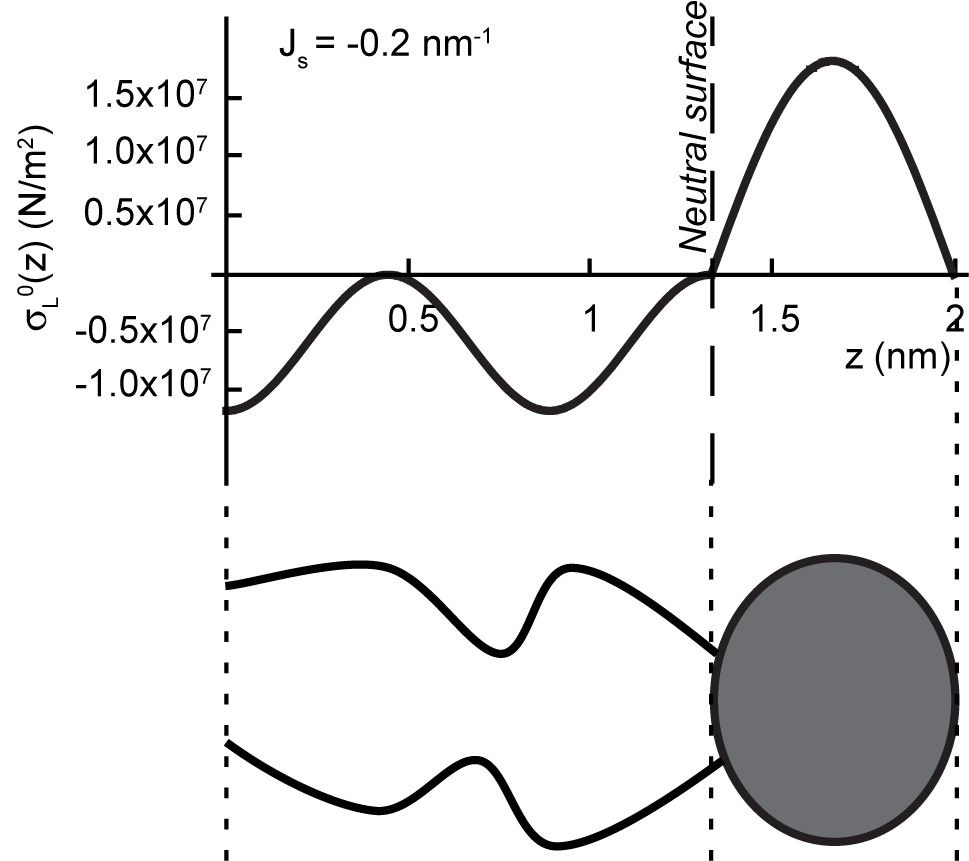

Supplement: Figure S2 — Lateral stress profile used in the computations. The depth modulation of the monolayer lateral stress profile used in the numerical computations as a result of varying the lipid composition is presented for a negatively curved monolayer with a spontaneous curvature of . (TIF) [file pcbi.1003556.s002.tif]
