# Supplementary material for: Sensing Membrane Stresses by Protein Insertions
Source: PLoS Comput Biol. 2014 Apr 10;10(4):e1003556. doi: 10.1371/journal.pcbi.1003556 (PMC3983069; doi:10.1371/journal.pcbi.1003556)
Supplement: Text S1 — This file contains detailed description of the model. Thermodynamic model of protein binding to membranes. Properties and the ways of generation of trans-membrane stress profile. (PDF) [file pcbi.1003556.s003.pdf]

Supporting Information Text S1

Thermodynamic model of protein binding to membranes

The goal of this section is to express the fraction of the total protein or protein domains (e.g. amphipathic helices) bound to the liposomes through the change of the membrane elastic energy resulting from the protein binding. To calculate the fraction of bound proteins to membranes we use the classical thermodynamic formalism in which the energy of protein binding to the membrane sets the equilibrium distribution between bound and unbound protein. We define a thermodynamic system consisting of N_l lipid molecules forming liposomes of a given size, N_w water molecules as solvent, and N_p proteins. The number of lipid molecules in solution is negligibly small compared to their number in liposomes. The number of the protein molecules in solution and in the liposome bound state will be denoted by N_p^f and N_p^b, respectively so that, $N_p^b + N_p^f = N_p$. The fraction of proteins bound to liposomes to the proteins remaining unbound is termed the binding constant, K_b,

$$K_b = \frac{N_p^b}{N_p^f} \tag{S1}$$

The mole fractions of proteins in solution and in the outer monolayers of the liposomes are, respectively,

$$c_f = \frac{N_p^f}{N_w} \text{ and } c_b = \frac{N_p^b}{N_l^{out}}, \tag{S2}$$

where N_l^{out} is the total number of lipid molecules in the outer membrane monolayers of all liposomes, and it is assumed that $N_p^f \ll N_w$, $N_p^b \ll N_l^{out}$. The number N_l^{out} is related to the total number of lipid molecules by

$$N_l^{out} = \frac{N_l}{2}(1 + J\,\delta), \tag{S3}$$

where J is the total curvature of the mid plane of the liposome membrane, and δ is the distance from the membrane mid plane to the monolayer neutral surface, where the lipid molecular areas are equal for the outer and inner membrane monolayers.

The mole fractions Eq. S2 can be related to the system energy through the requirement of the thermodynamic equilibrium between the bound and free protein. The chemical potentials of protein in solution and in the membrane can be written, respectively, as,

$$\begin{aligned} \mu_f &= \mu_f^0 + k_B T \log(c_f) \\ \mu_b &= \mu_b^0 + k_B T \log(c_b) \end{aligned} \tag{S4}$$

where μ_f^0 and μ_b^0 are the “standard” chemical potentials, which include all contributions to the free energy per protein molecule except for the contribution of the entropy of mixing, the latter taken into account by the terms logarithmic in the protein mole fraction; $k_B T \approx 4.21\ 10^{-21}\ Joule$ is the product of the Boltzmann constant and the absolute temperature.

Thermodynamic equilibrium requires equality of the protein chemical potentials, $\mu_b = \mu_f$, which leads to a relation between the mole fractions of the bound and free protein:

$$c_b = c_f \exp\left(-\frac{\varepsilon_{bind}}{k_B T}\right), \qquad \text{(S5)}$$

where $\varepsilon_{bind} = \mu_b^0 - \mu_f^0$ is the total free energy of binding of one insertion into the membrane. The binding energy can be presented as the sum of a contribution independent of the membrane stress, ε^0, and a contribution, ε_{el}, accounting for the change of the membrane elastic energy as a result of embedding of one insertion,

$$\varepsilon_{bind} = \varepsilon^0 + \varepsilon_{el} . \qquad \text{(S6)}$$

Based on Eqs. S5 and S6, we can present the ratio of the mole fractions of the bound and free protein as

$$\frac{c_b}{c_f} = \exp\left(-\frac{\varepsilon^0}{k_B T}\right) \exp\left(-\frac{\varepsilon_{el}}{k_B T}\right), \qquad \text{(S7)}$$

where the first term is a factor independent of the membrane stress.

The binding constant Eq. S1 is related to the mole fractions Eq. S2 by

$$K_b = \mathrm{B} \exp\left(-\frac{\varepsilon_{el}}{k_B T}\right), \qquad \text{(S8)}$$

where

$$\mathrm{B} = \frac{1}{2}\frac{N_l}{N_w}(1 + J\delta) \exp\left(-\frac{\varepsilon^0}{k_B T}\right), \qquad \text{(S9)}$$

is the stress-independent part that includes the correction for the different amount of lipids between the two monolayers of very small liposomes, where the curvature is $J = 2/R$.

The fraction of bound protein, $\Psi = \frac{N_p^b}{N_p}$, can be directly calculated from the binding constant as $\Psi = \frac{K_b}{1+K_b}$.

Properties and the ways of generation of trans-membrane stress profile

The trans-membrane stress profile is described by a function σ(z) [1], where z is the coordinate in the direction perpendicular to the monolayer plane measured, commonly, with respect to the so-called neutral plane lying close to the interface between the monolayer polar head and their hydrocarbon tails [2] (Fig. S1A). The product σ(z)dz gives the force acting on the unit length of an infinitesimal membrane element of thickness dz parallel to the membrane surface and located at the distance z from the neutral surface. According to the previous work, two kinds of stresses can develop with a fluid lipid monolayer: the lateral stress, $\sigma_L(z)$, acting along and isotropic within the monolayer plane, and transversal stress, $\sigma_T(z)$, directed perpendicularly to the monolayer plane. Here we consider the initial states of the membrane characterized by a certain lateral stress profile, $\sigma_L^0(z)$, but with a vanishing transversal stress,

$\sigma_T^0(z) = 0$, which implies that the monolayer elements can be, initially, stretched or compressed but there are no forces applied to the membrane and producing its global or local transverse stretch deformations. Embedding of an insertion can give rise to trans-monolayer distribution of the lateral, $u_L(z)$, and transversal, $u_T(z)$, strains of the membrane material. This results in contributions to the stress profiles related to the strains by the trans-monolayer profiles of the local elastic moduli: the lateral stretching modulus $\lambda_L(z)$, the transverse stretching modulus, $\lambda_T(z)$ (which is taken to be equal to the lateral stretching modulus), a coupling modulus, $\lambda_{LT}(z)$, and a transverse shear modulus, $\lambda_{SH}(z)$. Assuming the strains to be small, the stress profiles can be presented as [3]

$$\sigma_L(z) = \sigma_L^0(z) + \lambda_L(z)\, u_L(z) + \lambda_{LT}(z)\, u_T(z) \tag{S10}$$

$$\sigma_T(z) = \sigma_T^0(z) + \lambda_T(z)\, u_T(z) + \lambda_{LT}(z)\, u_L(z). \tag{S11}$$

The trans-monolayer distributions of elastic moduli is taken as

$$\lambda_L(z) = \lambda_T(z) = \lambda_{ST}(z) = \begin{cases} \lambda_{ST}^{chain}, & z \le z_n \\ \lambda_{ST}^{head}, & z > z_n \end{cases}, \tag{S12}$$

$$\lambda_{LT}(z) = \begin{cases} \lambda_{LT}^{chain}, & z \le z_n \\ \lambda_{LT}^{head}, & z > z_n \end{cases}, \tag{S13}$$

where $\lambda_{ST}^{chain} = 10^9$ N/m^2, $\lambda_{ST}^{head} = 4\times10^9$ N/m^2, $\lambda_{LT}^{chain} = 0.98\times10^9$ N/m^2, and $\lambda_{LT}^{head} = 3.93\times10^9$ N/m^2 [3]. The transverse shear modulus is constant and equal to $\lambda_{SH} = 1.5\times10^7$ N/m^2 everywhere except in the interface between the two monolayers, where the transverse shear modulus vanishes to allow inter-monolayer sliding.

The ways of generating the intra-membrane stress profile

The intra-membrane stresses $\sigma(z)$ can be produced by application to the membrane of external forces or by variations of the membrane lipid composition. To understand the essence of $\sigma(z)$ generation, it is convenient to consider the relationship between the internal stresses and the overall force factors determining the membrane stressed state, namely, the lateral tension γ and the bending moment τ. In most of the biologically relevant cases, the membrane monolayers can freely slip with respect to each other and, hence, undergo uncoupled deformations such that the monolayer γ and τ sum up to give those of the whole bilayer. Therefore, we focus in the following on the relationship for one monolayer.

The lateral tension of a flat monolayer is related to the lateral stress profile, $\sigma_L(z)$, by [4]

$$\gamma = \int \sigma_L(z) dz, \tag{S14}$$

It can be inferred from Eq. S14 that application to the monolayer of a stretching force generating a positive lateral tension $\gamma > 0$ results in an overall positive contribution to the trans-monolayer stress profile.

The monolayer bending moment τ can be expressed as the first moment of the trans-membrane lateral stress profile determined with respect to the neutral plane [4]

$$\tau = \int z\, \sigma_L(z) dz. \tag{S15}$$

As it follows from Eq. S15, the bending moment sets the relationship between the stresses above the neutral surface ($z > 0$) corresponding to the polar region and the stresses below the neutral plane ($z < 0$) corresponding to the monolayer hydrocarbon moiety. For example, application to the monolayer of a negative bending moment, $\tau < 0$, can modulate the distribution of the intra-monolayer stresses such that the stresses become negative ($\sigma(z) < 0$) above and positive ($\sigma(z) > 0$) below the neutral plane. Alternatively, the stresses may be negative both in the polar head and hydrocarbon chains sub-layers, but the effect of the former is stronger than that of the latter.

The bending moment, τ, and the corresponding intra-monolayer stress distribution, $\sigma_L(z)$, can be generated by both external forces and internal monolayer interactions. The external force factor producing the bending moment is a torque applied to the edge of an initially flat monolayer. The torque direction and value sets the sign and the value of τ.

The intra-monolayer factor contributing to the bending moment τ is the structure of the lipid molecules composing the monolayer. Insertion into the monolayer of molecules with bulkier polar heads and/or reduced hydrocarbon moieties modulates the intra-monolayer stress distribution such that the stress in the head group region ($z > 0$) becomes more negative compared to that in the hydrocarbon chain region ($z < 0$) (Fig. S1A). This results in a negative bending moment $\tau < 0$ (Fig. S1B). Lipids with a relatively small polar heads and large hydrocarbon moieties generate a positive bending moment, $\tau > 0$. An initially flat monolayer carrying an internal bending moment τ has a tendency to relax the bending moment by acquiring mean curvature, J.

Curvature of a monolayer is defined as positive, $J > 0$, if the monolayer bulges towards its polar heads and negative, $J < 0$, in the opposite case of bulging towards the hydrocarbon moiety.

For example, a monolayer having a negative bending moment, $\tau < 0$, tends to bulge towards the polar heads, hence, acquiring a positive curvature. This is accounted for by characterizing the monolayer by spontaneous curvature J_s related to the bending moment through

$$J_s = -\frac{\tau}{\kappa}, \qquad \text{(S16)}$$

where κ is the monolayer bending modulus [5]. A typical example of lipids generating a negative bending moment, $\tau < 0$, and, hence, a positive spontaneous curvature, $J_s > 0$, is lysolipids, whose hydrophobic moieties consist of one rather than two hydrocarbon tails. A positive bending moment, $\tau > 0$, corresponding to a negative spontaneous curvature, $J_s < 0$, is generated by such lipids as diacylglycerols (DAG) or phosphatidylethanolamines (PE) [6].

Based on Eqs. S15 and S16, we model the generation of the monolayer spontaneous curvature, J_s^{mon}, through a trans-monolayer lateral stress profile given by [7] (Fig. S2).

$$\sigma_L^0(z) = \begin{cases} \frac{18\,\kappa\pi^2 J_s^{mon}}{z_n(9h\pi^2+4z_n)}\left[1+\cos\left(\frac{3\pi z}{z_n}\right)\right], & z \le z_n \\ -\frac{9\,\kappa\pi^3 J_s^{mon}}{(h-z_n)(9h\pi^2+4z_n)}\sin\left(\pi\frac{z-z_n}{h-z_n}\right), & z > z_n \end{cases}, \qquad \text{(S17)}$$

References

1. Marsh D (2007) Lateral pressure profile, spontaneous curvature frustration, and the incorporation and conformation of proteins in membranes. Biophys J 93: 3884-3899.

2. Kozlov M, Winterhalter M (1991) Elastic moduli and neutral surface for strongly curved monolayers. Analysis of experimental results. Journal de Physique II 1: 1085-1100.
3. Campelo F, McMahon HT, Kozlov MM (2008) The hydrophobic insertion mechanism of membrane curvature generation by proteins. Biophys J 95: 2325-2339.
4. Helfrich W (1990) Elasticity and thermal undulations of fluid films of amphiphiles. Liquids at Interfaces 48.
5. Kozlov M, Helfrich W (1992) Effects of a cosurfactant on the stretching and bending elasticities of a surfactant monolayer. Langmuir 8: 2792-2797.
6. Zimmerberg J, Kozlov MM (2006) How proteins produce cellular membrane curvature. Nat Rev Mol Cell Biol 7: 9-19.
7. Illya G, Lipowsky R, Shillcock JC (2005) Effect of chain length and asymmetry on material properties of bilayer membranes. J Chem Phys 122: 244901.
